# Hydrate of Chloral in the Treatment of a Severe Case of Chorea

**Published:** 1870-08

**Authors:** J. S. Whitmire

**Affiliations:** Metamora, Ill.


ARTICLE IV.—*Hydrate of Chloral in the treatment of a severe case of Chorea.* By J. S. WHITMIRE, M.D., Metamora, Ill.

Having been reading up the therapeutic action of the Hydrate of Chloral as it has been published from time to time in our periodical medical literature, and having become satisfied of its powerful remedial agency in the treatment of our common neurotic diseases, I had determined, on the first occurring opportunity, to test its virtues. About the time that I had come to this determination, I was called in consultation with my brother and partner, Dr. Z. H. Whitmire, to see Miss A. H., aged 14 years. She had been afflicted with Chorea for four or five weeks, and had been under the usual treatment for the same length of time. The disease was first manifested in the twitching of the muscles of the face, which gradually extended to the left arm, at which time the parents naturally became alarmed and sought advice of my brother. He directed that her bowels be thoroughly evacuated, and then put her on Ferri. Mur. Tinc., Pot. Chlor. and Ammon. Mur., Spts. Nit. and water, three or four times per day. She was also given Pulv. Assafœtida and Quinine in powders four times per day. A hip bath was recommended at bedtime, and to have her surface well sponged every evening. Her bowels were kept in a soluble condition by mild laxatives, of which Aloes was an ingredient; this, with the hip bath, he supposed would bring on the catamenia, in case that this had anything to do with the exciting cause of her troubles, and, in any event, it could work no injury to his patient, even if it was not the cause of her malady. At the end of a week or ten days, this treatment proving of no avail, and his patient steadily growing worse, in addition to the above, he dry-cupped her spine every day from the nape of the neck to the lower dorsal vertebra, and as her nights were very restless, with disturbed sleep, he added a Dover's powder at bedtime to induce sleep. Ten or fifteen days more having elapsed without any amelioration of her symptoms, he directed her spine to be blistered, her feet bathed every night, a cup of Valerian tea to be given three or four times per day, with the continuance of the Chalybeate Mixture, and her condition persistently growing worse. About this time I was called with him to see her. I found her the most pitiable object that I ever beheld. By this time she could neither sit, lie nor stand, but was continually in motion, every muscle in her body partaking of irregular motion; her face drawn into the most ludicrous grimaces; her head, limbs and body in constant motion; and unable to articulate a single syllable. She had to be fed by her friends, and very often swallowed both her food and medicine with great difficulty. She had slept but very little for more than ten days, and even when she would get into a disturbed rest, she would awaken in the most terrible alarm, and scream and cry to the extent of her ability, and listen to no attempt that was made to quiet her, till she would be completely exhausted, so that she was in imminent danger, at any moment, of going into general convulsions. She had become anemic and very much emaciated, so that the vital forces were nearly exhausted, and was now entirely incapable of locomotion. And, while I am aware of the fact that but few die of this, without some intercurrent disease, I am convinced that the VITAL SPARK in this case must soon have become extinguished had not an abatement of the disease speedily taken place. I prescribed Griffith's Antilithic Mixture, prepared with Aqua Camphoræ, instead of Aqua Rosæ, as directed by the pharmacopœia, and added an ounce of Sugar and a half-ounce of Tinct. Guiaac. to an eight ounce mixture, of which she took one teaspoonful, in water, three times per day. I continued the Pulv. Assafœtida and Quinine four times per day, and, at night, to procure sleep, of which she had been so long deprived, I administered from ten to twelve grains of Hyd. Chloral, dissolved in two ounces of water. When it was necessary, her bowels were opened with a mild laxative pill, and her diet consisted of beef tea, all she could be induced to swallow, and soft boiled eggs, till convalescence was beginning to be established, after which she was allowed solid food. For fear of accident, after having given her the first dose of Chloral, I remained at her bed-side till she went to sleep, which was probably not to exceed half an hour, and I then remained an hour longer to observe the effect after she should be completely under its influence. At the end of that time I was satisfied that she would rest the balance of the night, so that I left her without the least apprehension of danger from the medicine. In the morning I found that she had slept SEVEN hours as tranquilly as a babe, having awakened once or twice during the time, but dropped into quiet slumber in a moment after. She was now quieter than she had been for some time, and on every returning evening I repeated the same dose of Chloral till she was clearly convalescent, which was seven or eight days, after which the dose was gradually lessened for a week more, and then abandoned altogether. The Chalybeate Mixture, Assafœtida and Quinine, was continued till her health and strength were perfectly restored, which was probably four or five weeks from the time that the Hyd. of Chloral was first used.

In this case, the Chalybeates, bitter tonics, and anti-spasmodics were clearly indicated, and, as far as they went, were just what the child needed, but they were not sufficient within themselves to fill all the indications of treatment in this particular case—for none of them would, or did, produce rest, sleep and tranquillity—just the three things most necessary to produce a change in her condition. The only thing necessary, in the whole catalogue of remedies, to put my patient upon praying grounds, in this erratic disease, along with the other treatment, was something that would induce sleep, without, in the least, disturbing any of the natural functions of life. This was found in the Hyd. of Chloral, and without it, or its equivalent, she must have succumbed to the exhaustion produced by the incessant involuntary motion to which she was subjected. But the rest and sleep produced by this agent, permitted the vital forces to resume their wonted power, and jaded nature to again build up the wasted tissues and resume their rounded form.

JUNE 22d, 1870.

